# Application of Machine Learning Classifier to *Candida auris* Drug Resistance Analysis

**DOI:** 10.3389/fcimb.2021.742062

**Published:** 2021-10-15

**Authors:** Dingchen Li, Yaru Wang, Wenjuan Hu, Fangyan Chen, Jingya Zhao, Xia Chen, Li Han

**Affiliations:** ^1^ Department of Disinfection and Infection Control, Chinese People’s Liberation Army (PLA) Center for Disease Control and Prevention, Beijing, China; ^2^ School of Mathematics and Statistics, Shaanxi Normal University, Xi’an, China

**Keywords:** *Candida auris*, phylogenetic analysis, drug resistance, whole genome sequencing, machine learning, antifungal drugs, ergosterol pathway

## Abstract

*Candida auris (C. auris)* is an emerging fungus associated with high morbidity. It has a unique transmission ability and is often resistant to multiple drugs. In this study, we evaluated the ability of different machine learning models to classify the drug resistance and predicted and ranked the drug resistance mutations of *C. auris*. Two *C. auris* strains were obtained. Combined with other 356 strains collected from the European Bioinformatics Institute (EBI) databases, the whole genome sequencing (WGS) data were analyzed by bioinformatics. Machine learning classifiers were used to build drug resistance models, which were evaluated and compared by various evaluation methods based on AUC value. Briefly, two strains were assigned to Clade III in the phylogenetic tree, which was consistent with previous studies; nevertheless, the phylogenetic tree was not completely consistent with the conclusion of clustering according to the geographical location discovered earlier. The clustering results of *C. auris* were related to its drug resistance. The resistance genes of *C. auris* were not under additional strong selection pressure, and the performance of different models varied greatly for different drugs. For drugs such as azoles and echinocandins, the models performed relatively well. In addition, two machine learning algorithms, based on the balanced test and imbalanced test, were designed and evaluated; for most drugs, the evaluation results on the balanced test set were better than on the imbalanced test set. The mutations strongly be associated with drug resistance of *C. auris* were predicted and ranked by Recursive Feature Elimination with Cross-Validation (RFECV) combined with a machine learning classifier. In addition to known drug resistance mutations, some new resistance mutations were predicted, such as Y501H and I466M mutation in the *ERG11* gene and R278H mutation in the *ERG10* gene, which may be associated with fluconazole (FCZ), micafungin (MCF), and amphotericin B (AmB) resistance, respectively; these mutations were in the “hot spot” regions of the ergosterol pathway. To sum up, this study suggested that machine learning classifiers are a useful and cost-effective method to identify fungal drug resistance-related mutations, which is of great significance for the research on the resistance mechanism of *C. auris*.

## Introduction

*Candida auris* (*C. auris*) is an emerging fungal pathogen first isolated from the external ear canal of a 70-year-old female inpatient in Tokyo hospital (Satoh et al., 2009). *C. auris* can persist for weeks in a nosocomial environment, and survive high-end disinfections, thus presenting a serious global health threat (Chaabane et al., 2019; Du et al., 2020). To date, *C. auris* outbreak has been reported in more than 30 countries worldwide (Rhodes et al., 2018; Tian et al., 2018; Escandon et al., 2019; Rhodes and Fisher, 2019). *C. auris*, also known as “super fungus”, is a multidrug-resistant species associated with high mortality (Wang et al., 2018).

So far, four specific clades of *C. auris* have been identified by phylogenetic analysis based on whole-genome sequencing (WGS): South Asia (Clade I), East Asia (Clade II), South Africa (Clade III), and South America (Clade IV). A potential fifth clade of Iranian origin was described by few studies (Chow et al., 2019; Di Pilato et al., 2021). All clades are characterized by distinct single nucleotide polymorphisms (SNPs), highlighting this pathogen’s independent and worldwide emergence (Lockhart et al., 2017). Except for Clade II, the other three clusters have been associated with an outbreak of invasive infection and multiple resistance. Clade II is predominantly an ear canal infection, and presents either single fluconazole resistance or susceptible (Kwon et al., 2019; Welsh et al., 2019).

Clinically, invasive fungal infections are usually treated with three classes of antifungal agents: echinocandins, azoles, and polyenes (ElBaradei, 2020). Fluconazole (FCZ) resistance is the most common. Resistance to other azoles like voriconazole (VCZ), itraconazole (ICZ), and posaconazole (PZ) might vary (Montoya et al., 2019; ElBaradei, 2020).

Ergosterol is a key component of the fungal cell membrane. In *Candida*, ergosterol is mediated by lanosterol 14-alpha-demethylase (*ERG11*), which is involved in an important step in the biosynthesis of ergosterol. Antifungal agents effectively inhibit ergosterol biosynthesis by inhibiting the enzyme’s function, thereby compromising membrane integrity (Sanglard et al., 1998). Different mechanisms, including mutations in the *ERG11* gene, overexpression of the ATP-binding Cassette (*ABC*) exogenous pump transporter, which is encoded by the *CDR1* gene, and duplication and overexpression of the *ERG11* gene, contribute to the reduction of the sensitivity of *C. auris* to azole drugs (Puri et al., 1999; de Micheli et al., 2002; Coste et al., 2004; Cannon et al., 2009; Noel, 2012; Spampinato and Leonardi, 2013; Medici and Del Poeta, 2015; Nami et al., 2019; Bing et al., 2020). Point mutations in the *ERG11* gene, associated with FCZ resistance in *Candida albicans*, are also one of the mechanisms of FCZ resistance in *C. auris*. Point mutations in *ERG11* can reduce the azole sensitivity of *Candida*, particularly in the “hot spots” located between 105-165, 266-287, and 405-488 (Lamb et al., 1995; Sanglard et al., 1998; Mellado et al., 2004; Vandeputte et al., 2012). Moreover, Lockhart et al. described three major mutations in *ERG11* that influence FCZ resistance, namely, F126T, Y132F, and K143R (Lockhart et al., 2017). Furthermore, Healey et al. found that Y132F mutations significantly reduce the sensitivity of *C. auris* to azole drugs. Also, it has been reported that these mutations are associated with geographic cues, with mutations leading to Y132F and K143R associated with isolates belonging to South Asian and South American groups (Healey et al., 2018). In addition, Rybak et al. reported new mutations on the zinc-cluster transcription factor-encoding gene (*TAC1B*) associated with FCZ resistance (Rybak et al., 2020). This study showed that mutations on *TAC1B* could be produced rapidly *in vitro* after exposure to FCZ. Most FCZ-resistant isolates have many drug-related *TAC1B* mutations in a specific global lineage or group of *C. auris*, and the identification of new resistance determinants has significantly increased the understanding of clinical antifungal resistance in *C. auris* (Rybak et al., 2020).

*C. auris* resistance to echinocandins is less common. Caspofungin (CSF), micafungin (MCF), and anidulafungin (AND) are often recommended as first-line treatments for candidemia (ElBaradei, 2020). *In vitro* studies have demonstrated that CSF and AND have a certain inhibitory effect on the growth of *C. auris* (Dudiuk et al., 2019). Interestingly, one study reported that among all echinocandins, micafungin has the highest inhibitory effect against *C. auris* (Kordalewska et al., 2018).

Echinocandins inhibit the 1, 3-beta-D-glucan synthetase required for cell wall synthesis, encoded by the genes *FKS1* and *FKS2*. Several mutations (“hot spots 1 and 2”) in the *FKS1* and *FKS2* genes in *Candida albicans* and other non-auris *Candida* species have been associated with the echinocandins resistance. In the *FKS1* gene of *C. albicans*, these “hot spots” lie between the amino acids 641-649 and 1,345-1,365 (Park et al., 2005). Resistance to the echinocandins involves mutations in the *FKS1* gene, with changes in the hot spot 1 region leading to amino acid substitution from serine to proline at 639 (S639P) (Biagi et al., 2019). Moreover, a multicenter study in India reported another mutation in the same position 639 of the *FKS1* gene, involving a change from serine to phenylalanine (S639F or S639Y) (Chowdhary et al., 2018). Sharma et al. also found *FKS2* in a single copy of the *C. auris* genome; yet, no mutation associated with echinocandins resistance has been found in this gene (Sharma et al., 2016; Chaabane et al., 2019).

Among polyenes, *C. auris* and *C. lusitaniae* have shown high resistance to amphotericin B (AmB). However, the molecular mechanism of polyene drug resistance is not clear (ElBaradei, 2020) and more research may be needed to reveal how non-synonymous mutations promote resistance to AmB in *C. auris* (Escandon et al., 2019). Kordalewska and Perlin suggested that resistance to AmB is regulated at the transcriptional level rather than mutations (Kordalewska and Perlin, 2019).

Predictive models based on machine learning can explore multiple associations between genetic variations. Machine learning is the scientific discipline that focuses on how computers learn from data (Deo, 2015). As an essential component in artificial intelligence (AI), it has been integrated into many fields, such as data generation, analytics and knowledge mining (Handelman et al., 2018; Patel et al., 2020). Several previous studies have used machine learning algorithms to predict microbial resistance. For example, Zhang et al. collected 161 strains of *Mycobacterium tuberculosis* (MTB) from China and used logistic regression and random forest to find and predict new genes associated with drug resistance of seven drugs (Zhang et al., 2013). Furthermore, using a more geographically diverse data set, Farhat et al. studied the performance of the random forest algorithm based 1,397 isolates (Farhat et al., 2016). Her et al. proposed a pan-genome-based method to characterize antibiotic-resistant microbial strains; the method was tested on *Escherichia coli*. The drug resistance gene was predicted by identifying the core and accessory gene clusters on *Escherichia coli* pan-genomic (Her and Wu, 2018). In addition, Yang et al. considered 1,839 bacterial isolates from the UK and compared the performance of more machine learning classifiers, including Logistic Regression, Support Vector Classifier (based on linear and Gaussian kernel functions), product-of-marginals model (PM), Random Forest, gradient tree boosting (GBT), and Adaboost. Finally, mutations associated with drug resistance of MTB ranked and were predicted (Yang et al., 2018; Kouchaki et al., 2019). However, most of the microbes studied were bacteria, while only a few studies applied this method to study fungi. Moreover, currently, there are no studies on the classification of fungi drug resistance and the evaluation of drug resistance mutations by mathematical models.

In this study, we collected *C. auris* isolates from different countries or regions, analyzed their whole genome sequencing data, constructed the phylogenetic relationship, evaluated the ability of different machine learning models to classify the drug resistance, and predicted and ranked the drug resistance mutations of *C. auris*.

## Materials and Methods

### WGS and Pre-processing

As of April 2020, the whole genome sequencing (WGS) data of *C. auris* published by the European Bioinformatics Institute (EBI, https://www.ebi.ac.uk/) has 796 isolates in total. Among them, 356 strains have undergone antifungal susceptibility testing. According to these results, resistant or susceptible strains were determined according to the Clinical and Laboratory Standards Institute (CLSI) guidelines.

In this study, WGS data of 356 strains containing drug resistance information on the EBI website were collected, and two strains named C1921 and C1922, which showed FCZ resistance from the Chinese PLA Center for Disease Control & Prevention were combined (Chen et al., 2018). This study involved WGS data of 358 C*. auris* strains (see **Supplementary Materials File**), all of which were sequenced using Illumina sequencing technology platform; the sequencing data obtained were double-ended WGS data in FASTQ data format. The drug resistance of 358 strains above was collected, including fluconazole, itraconazole, voriconazole, posaconazole, amphotericin B, micafungin, anifenqine and caspofunqine. The statistics of drug resistance of the strains are shown in **Table 1**.

**Table 1:** Classification of all *C. auris* strains’ drug-resistant phenotypes.

Drugs	FCZ	AmB	MCF	VCZ	ICZ	PZ	AND	CSF
**Resistant**	254	80	24	19	10	39	3	3
**Susceptible**	104	273	321	104	108	70	113	119
**Missing**	0	5	13	235	240	249	242	236

WGS data of 358 C*. auris* strains were collected and analyzed using the following steps: FastQC (http://www.bioinformatics.babraham.ac.uk/projects/fastqc/) checked the data quality of each strain’s sequence and divided the data according to different types of sequencing adapters for quality control [Trimmomatic (Bolger et al., 2014)]. All data were aligned and sorted with the reference strain B8441 using Bwa-0.7.17 (Munoz et al., 2018). Duplicates in the file were marked using MarkDuplicates module in GATK (DePristo et al., 2011) v4.1.4.1, and were ignored during the mutation detection. In BaseRecalibrator, 246,258 sites were jointly detected by GATK HaplotypeCaller and Bcftools (Li et al., 2009) mpileup, which were finally used as SNP reference sets.

The recalibration of base mass values mainly involved two steps: GATK BaseRecalibrator and GATK ApplyBQSR. Then, the mutation detection was performed by GATK HaplotypeCaller. Finally, VCFtools (Danecek et al., 2011) software was used to filter the samples and detection sites, respectively. Two samples with high deletion rates (max-missing ≥ 50%) (SRR10461133 and SRR10461145) were removed from the filtering of the samples. The sites with minQ ≤ 30, max-missing ≥ 0.5, mac ≤ 3, and minDP ≤ 3 were deleted, respectively, using VCFtools, and the number of sites after filtering was 229,262. The filtered files were annotated using SNPEff (Cingolani et al., 2012), and the annotated files were used for phylogenetic analysis and machine learning resistance analysis. Three antifungals (FCZ, MCF and AmB) and point mutations (Y132F, K143R and F126L in ERG11, S639Y/S639F and S639P in FKS1) was also depicted in the phylogenetic NJ tree. This process is shown in **Figure S1** and **Table S1**.

### Selection and Extraction of Gene Sets

A total of 229,262 SNP mutation sites were found in 358 *C. auris* isolates. Candidate genes that may have a strong correlation with drug resistance of *C. auris* in previous studies were selected; this was performed in order to reduce its dimension, facilitate machine learning classification, eliminate redundant sites, and improve the accuracy of the analysis for the complex dimension. In addition, only missense mutations were extracted for further analysis since they accounted for only a small part of the original mutations, but affected the type of amino acids, i.e., the function of proteins.

Three candidate gene sets were selected in this study (Lockhart et al., 2017; Munoz et al., 2018; Chaabane et al., 2019; Rybak et al., 2020) (**Table S2**). F3 set included genes that were previously reported to be associated with drug resistance and may contain determinants of drug resistance information in *C. auris*; F2 set was a list of seven genes specific in C*. auris*, which have been associated with drug resistance in *C. albicans*, but are highly conserved in *C. auris* (Munoz et al., 2018). F1 set combined the F2 and F3 genes. All the missense mutations were extracted in the three gene sets and filtered. The samples and sites with too many missing values for each set were deleted, and the dimension of the data set after processing the missing values (samples × mutations) was respectively: F1: 350 x 579; F2: 353 x 202; F3: 352 x 377.

### Machine Learning Algorithms

Two algorithms were designed by using Python 3.8.4 (https://www.python.org/downloads/): the classifier on the balanced test set and on the imbalanced test set (**Figures S2**, **S3**). The F1, F2, and F3 sets were used as the classification feature sets, and the drug resistance of *C. auris* was taken as the classification target. Ten machine learning classifiers (**Table S3**), Logistic Regression (LR), Support Vector Classifier (SVC, including SVC RBF and SVC linear), K-Nearest Neighbors (KNN), Decision Tree (DT), Ensemble Learning (including RandomForest, AdaBoost and GradientBoosting), and Naive Bayes (including BernoulliNB and GaussianNB) were used to build the model (Breiman, 1996; Breiman, 2001) by using Python 3.8.4. For AdaBoost, the Decision Tree Classifier was the base estimator whose number was 200 and the max depth was 1. There were 100 trees set in the random forest classifier. The neighbor was 5 (the value of K) for the KNN classifier. In both algorithms, principal component analysis (PCA) was used to reduce dimensionality based on retaining 99% of the original information. The number of principal components after dimension reduction with PCA method when 99% of the variance is retained is in supplementary material **Table S4**. Upsampling and downsampling were mainly adopted to balance the data set and repeated sampling 100 times. Downsampling means, for a dataset from the majority classification, creating a new subset with the same sample number as the minority classification from the original set by random sampling. Upsampling means, for a dataset from the minority classification, creating a new dataset with the same sample number as the majority classification from the original set by random sampling. The data were divided into test set and training set according to 5-fold cross-validation (5-CV), which accounted for 20% and 80%, respectively. The model parameters were adjusted on a training set, and the model was retrained using 5-CV. Finally, the model was evaluated on the test set. The area under the ROC (the Receiver operating characteristic curve) curve (AUC), was used as evaluation standard of a model’s performance. A classifier with a larger AUC (closer to 1.0) performed better.

### Recursive Feature Elimination With Cross-Validation

The Recursive Feature Elimination with Cross-validation (RFECV) functions in Python’s Scikit-Learn established in mutation sequencing were based on the F1 data set, which contained all candidate genes selected before machine learning modeling. All features were standardized before ranking, and the training model was the classifier above. The standardized method used was StandardScaler() function in Python. The number of features discarded in each iteration was 1, indicating elimination one by one, and the model was built repeatedly through 5-CV.

## Results

### Phylogenetic Analysis of *Candida auris*

**Figure 1:**
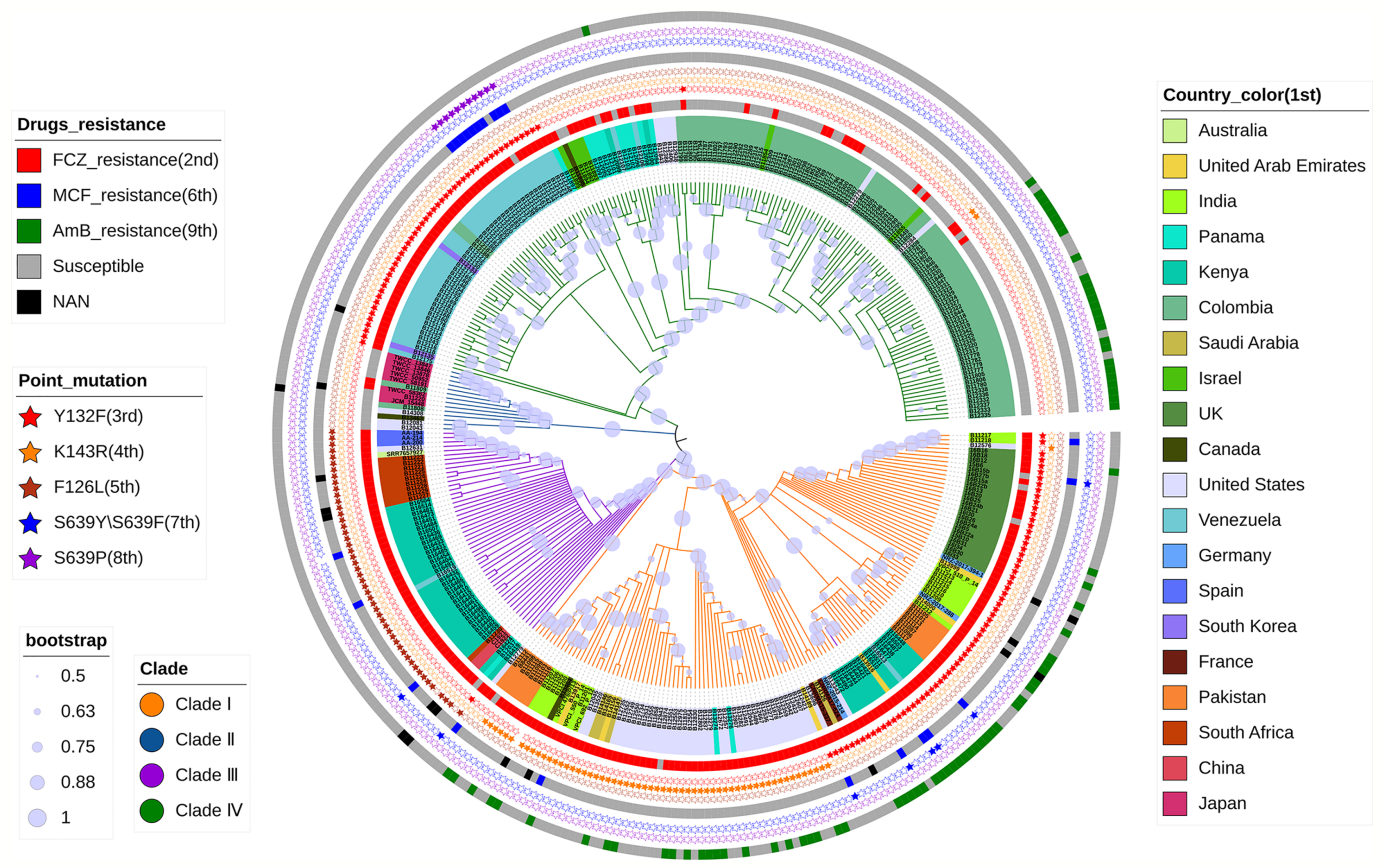
Phylogenetic NJ tree based on WGS of *C. auris*. The tree describes the phylogeny of 356 strains of *C. auris* from different regions, divided into four clades. The 1^st^ to 9^th^ indicates a concentric circle from the inner-most to the outer-most, respectively. It also shows the correlation between the resistance of these strains to FCZ, AmB, and MCF and the reported point mutations with Y132F, K143R, and F126L in *ERG11*, S639Y/S639F, and S639P in *FKS1*.

Phylogenetic NJ-tree was constructed using MEGA-X (Kumar et al., 2018), and boostrap test was repeated 500 times. Then, the phylogenetic tree was annotated using the iTOL online tool (https://itol.embl.de/). The phylogenetic NJ tree was divided into four clades starting from the root (**Figure 1**): Clade I (orange), Clade II (blue), Clade III (purple), and Clade IV (green), which was consistent with the conclusions reported in previous literatures (Lockhart et al., 2017). The clustering results are shown in **Table S5**. However, strain B16401 (SRR10852068, Kenya) was assigned to Clade I in this study; in a previous study, strain B16401 was assigned to Clade III (Chow et al., 2020). In the NJ tree, C1921 and C1922 from our laboratory were in Clade III, which was consistent with the phylogenetic tree constructed using Internal Transcribed Spacer (ITS) and D1/D2 Large Ribosomal Subunit Region previously (Chen et al., 2018). In addition, the mutations associated with azoles and echinocandins resistance detected were consistent with the previous conclusions (Chow et al., 2020). According to these results, F126L mutation in lanosterol 14-alpha-demethylase *ERG11* occurred in C1921 and C1922 strains, which is closely related to their FCZ resistance observed in clinical practice. It was also shown that the phylogenetic tree constructed by the drug-resistant gene set F3 was very similar to the phylogenetic tree constructed by the WGS of *C. auris*, and there was no difference in the clustering results of the strains (**Figure S4**), indicating that the evolution of *C. auris* resistance genes was consistent with the overall evolution of the strains (at the level of the whole genome). It was speculated that the resistance genes of *C. auris* were not under additional strong selection pressure, which may be related to the clinical use of drugs.

### Evaluation of Classification Models

The performances of machine learning classifiers, constructed by the two algorithms described above on F1, F2, and F3, were evaluated and compared by several evaluation methods. The best model for each set and drug was listed in **Table 2**. For most drugs, the evaluation results on the balanced test set were better than on the imbalanced test set. The classifiers established using two algorithms achieved better results for azoles, like FCZ, ICZ and VCZ, since their AUC values were above 0.9. However, compared with other drug models, the evaluation results of AmB needed to be improved; we speculated that this might be closely related to the selection of candidate genes. For well-studied drugs (azoles and echinocandins), the selected three gene sets contained more information about determinants associated with drug resistance, but there were few determinants of polyenes resistance.

**Table 2:** Model evaluation results under two different algorithms: on the balanced test set (the upper table) and on the imbalanced test set (the lower table).

The best model	AUC value	Sensitivity	Specificity	Accuracy	Recall	F1 score	Threshold
F1AmB_AdaBoost_Upsampling	0.9507 ± 0.0082	0.9504 ± 0.0178	0.8554 ± 0.0096	0.9019 ± 0.0140	0.9502 ± 0.0179	0.9066 ± 0.0138	0.5012 ± 0.0002
F2AmB_KNeighbors_Upsampling	0.8719 ± 0.0249	0.8057 ± 0.0671	0.8025 ± 0.0093	0.7992 ± 0.0323	0.8057 ± 0.0671	0.7920 ± 0.0406	0.7187 ± 0.0532
F3AmB_RF_Upsampling	0.9026 ± 0.0285	0.8711 ± 0.0465	0.7818 ± 0.0395	0.8227 ± 0.0435	0.8710 ± 0.0465	0.8303 ± 0.0423	0.5851 ± 0.0316
F1MCF_KNeighbors_Upsampling	0.9971 ± 0.0007	0.9926 ± 0.0060	0.9908 ± 0.0068	0.9865 ± 0.0072	0.9926 ± 0.0060	0.9867 ± 0.0071	0.9561 ± 0.0247
F2MCF_KNeighbors_Upsampling	0.9648 ± 0.0127	0.9691 ± 0.0130	0.8882 ± 0.0345	0.9078 ± 0.0253	0.9691 ± 0.0129	0.9140 ± 0.0228	0.8026 ± 0.0281
F3MCF_RF_Upsampling	0.9914 ± 0.0044	0.9401 ± 0.0365	0.9825 ± 0.0100	0.9604 ± 0.0231	0.9395 ± 0.0370	0.9586 ± 0.0245	0.8755 ± 0.0564
F1FCZ_RF_Upsampling	0.9908 ± 0.0043	0.9542 ± 0.0138	0.9769 ± 0.0173	0.9615 ± 0.0156	0.9483 ± 0.0129	0.9609 ± 0.0155	0.6040 ± 0.0136
F2FCZ_AdaBoost_Upsampling	0.9621 ± 0.0048	0.9129 ± 0.0144	0.9860 ± 0.0061	0.9502 ± 0.0110	0.9120 ± 0.0143	0.9478 ± 0.0118	0.5436 ± 0.0217
F3FCZ_RF_Upsampling	0.9787 ± 0.0076	0.9380 ± 0.0124	0.9703 ± 0.0207	0.9533 ± 0.0171	0.9377 ± 0.0126	0.9527 ± 0.0170	0.7207 ± 0.0567
F1VCZ_KNeighbors_Upsampling	0.9690 ± 0.0094	0.9710 ± 0.0077	0.9089 ± 0.0243	0.9262 ± 0.0157	0.9704 ± 0.0078	0.9306 ± 0.0147	0.8061 ± 0.0567
F2VCZ_LR_Downsampling	0.9381 ± 0.0025	0.8924 ± 0.0030	0.9477 ± 0.0009	0.8753 ± 0.0016	0.8567 ± 0.0023	0.8721 ± 0.0021	0.5064 ± 0.0016
F3VCZ_AdaBoost_Upsampling	0.9485 ± 0.0056	0.9959 ± 0.0048	0.8587 ± 0.0052	0.9272 ± 0.0043	0.9959 ± 0.0048	0.9320 ± 0.0040	0.5016 ± 0.0003
F1PZ_DecisionTree_Upsampling	0.9251 ± 0.0429	0.9099 ± 0.0479	0.8347 ± 0.0577	0.8689 ± 0.0534	0.9087 ± 0.0485	0.8735 ± 0.0522	0.7408 ± 0.0563
F2PZ_RF_Upsampling	0.7872 ± 0.0605	0.7496 ± 0.1100	0.8173 ± 0.0116	0.7822 ± 0.0616	0.7496 ± 0.1100	0.7628 ± 0.0807	0.7732 ± 0.0515
F3PZ_RF_Upsampling	0.8919 ± 0.0472	0.9057 ± 0.0660	0.8010 ± 0.0455	0.8350 ± 0.0571	0.9057 ± 0.0660	0.8442 ± 0.0566	0.7275 ± 0.0626
F1ICZ_KNeighbors_Upsampling	0.9651 ± 0.0099	0.9945 ± 0.0036	0.9220 ± 0.0220	0.9528 ± 0.0106	0.9944 ± 0.0036	0.9563 ± 0.0095	0.8884 ± 0.0332
F2ICZ_AdaBoost_Downsampling	0.9874 ± 0.0032	1.0000 ± 0.0000	0.9749 ± 0.0064	0.9800 ± 0.0048	1.0000 ± 0.0000	0.9840 ± 0.0038	1.0000 ± 0.0000
F3ICZ_BernoulliNB_Downsampling	0.9701 ± 0.0014	1.0000 ± 0.0000	0.9000 ± 0.0000	0.9500 ± 0.0000	1.0000 ± 0.0000	0.9600 ± 0.0000	0.9995 ± 0.0000
The best model	AUC value	Sensitivity	Specificity	Accuracy	Recall	F1 score	Threshold
F1AmB_RF_Downsampling	0.9136 ± 0.0144	0.7365 ± 0.0935	0.9024 ± 0.0154	0.8565 ± 0.0116	0.7335 ± 0.0945	0.6908 ± 0.0472	0.6003 ± 0.0018
F2AmB_GB_Downsampling	0.8008 ± 0.0033	0.1980 ± 0.0278	0.9997 ± 0.0005	0.8118 ± 0.0084	0.1980 ± 0.0278	0.3214 ± 0.0410	0.9759 ± 0.0264
F3AmB_RF_Downsampling	0.8116 ± 0.0244	0.4220 ± 0.0278	0.9230 ± 0.0325	0.8009 ± 0.0226	0.4150 ± 0.0300	0.4904 ± 0.0223	0.5949 ± 0.0076
F1MCF_SVC_RBF_Upsampling	0.9807 ± 0.0162	0.9186 ± 0.0764	0.9911 ± 0.0033	0.9825 ± 0.0034	0.9186 ± 0.0764	0.8540 ± 0.0433	0.9516 ± 0.0188
F2MCF_GB_Upsampling	0.7565 ± 0.0179	0.6240 ± 0.0698	0.8086 ± 0.0128	0.7846 ± 0.0086	0.6240 ± 0.0698	0.2615 ± 0.0291	0.6777 ± 0.0155
F3MCF_LRL2_Upsampling	0.9510 ± 0.0089	0.8121 ± 0.0485	0.9750 ± 0.0065	0.9010 ± 0.0064	0.8121 ± 0.0485	0.5121 ± 0.0215	0.9735 ± 0.0136
F1FCZ_RF_Downsampling	0.9593 ± 0.0043	0.9707 ± 0.0029	0.8700 ± 0.0213	0.9312 ± 0.0112	0.9695 ± 0.0030	0.9527 ± 0.0075	0.5901 ± 0.0097
F2FCZ_RF_Upsampling	0.9314 ± 0.0095	0.9053 ± 0.0065	0.9294 ± 0.0154	0.9076 ± 0.0087	0.9026 ± 0.0077	0.9328 ± 0.0065	0.6716 ± 0.0356
F3FCZ_RF_Downsampling	0.9531 ± 0.0090	0.2122 ± 0.0792	0.8966 ± 0.0412	0.9049 ± 0.0080	0.9225 ± 0.0101	0.9321 ± 0.0050	0.5903 ± 0.0207
F1VCZ_RF_Downsampling	0.9341 ± 0.0441	0.8222 ± 0.0628	0.9270 ± 0.0462	0.8921 ± 0.0340	0.8218 ± 0.0631	0.7092 ± 0.0693	0.7420 ± 0.0211
F2VCZ_LRL2_Upsampling	0.9136 ± 0.0498	0.9204 ± 0.0699	0.8978 ± 0.0377	0.8731 ± 0.0509	0.9202 ± 0.0702	0.7159 ± 0.1058	0.5389 ± 0.0053
F3VCZ_SVC_Linear_Upsampling	0.9434 ± 0.0300	0.9211 ± 0.0693	0.9247 ± 0.0198	0.8594 ± 0.0211	0.8775 ± 0.0760	0.6554 ± 0.0550	0.7265 ± 0.0689
F1PZ_RF_Downsampling	0.7846 ± 0.0270	0.5016 ± 0.0285	0.8447 ± 0.0156	0.7090 ± 0.0117	0.4967 ± 0.0285	0.5429 ± 0.0211	0.5986 ± 0.0088
F2PZ_LRL2_Downsampling	0.6595 ± 0.0296	0.3707 ± 0.0148	0.8793 ± 0.0290	0.6950 ± 0.0201	0.3707 ± 0.0148	0.4596 ± 0.0312	0.5617 ± 0.0386
F3PZ_RF_Downsampling	0.6737 ± 0.0346	0.4131 ± 0.0409	0.8787 ± 0.0279	0.6831 ± 0.0045	0.4068 ± 0.0366	0.4712 ± 0.0172	0.6173 ± 0.0275
F1ICZ_KNeighbors_Upsampling	0.9696 ± 0.0168	0.9585 ± 0.0261	0.9388 ± 0.0224	0.9404 ± 0.0204	0.9585 ± 0.0261	0.7479 ± 0.0457	0.7861 ± 0.0461
F2ICZ_LRL2_Upsampling	0.9375 ± 0.0112	1.0000 ± 0.0000	0.8750 ± 0.0224	0.8838 ± 0.0231	1.0000 ± 0.0000	0.6040 ± 0.0382	0.8403 ± 0.0281
F3ICZ_BernoulliNB_Upsampling	0.9657 ± 0.0144	1.0000 ± 0.0000	0.9276 ± 0.0226	0.9323 ± 0.0207	1.0000 ± 0.0000	0.7310 ± 0.0477	0.9997 ± 0.0000

The number after ±/indicates the standard deviation after 100 repeated samplings.

**Figure 2:**
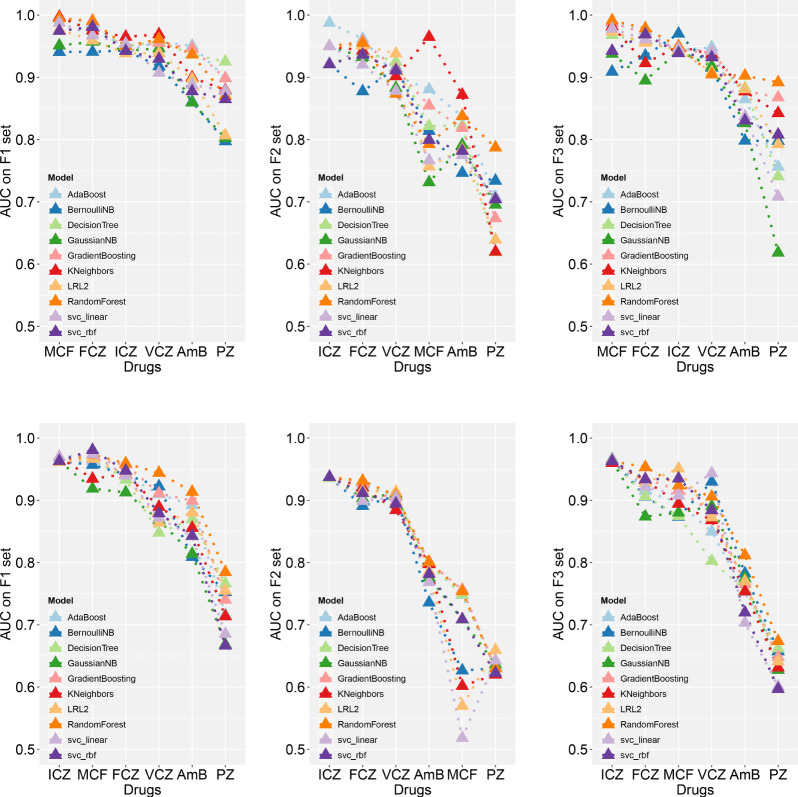
Comparison of the best AUC values using different machine learning classifiers.The best models on the balanced (the upper three) and imbalanced (the lower three) test set are shown, respectively. Please see supplementary materials **Table S6** for detailed evaluation results.

The model with the highest AUC value was extracted and compared (**Figure 2** and **Table S6**). Random forest, logistic regression, and K-nearest neighbors ranked in the top and for several times. Under two algorithms, the classifier models performed well on F1 for all drugs, of which the AUC values were above 0.85. While on F2 and F3, classifiers performed well only on some drugs; for example, models performed well on F2 for azoles like FCZ, ICZ, and VCZ, and they performed well on F3 for MCF, but they all had poor classification effect on AmB and PZ. It may be that the correlation between the three sets and classification targets was not very strong, and the information collected for these two drugs was insufficient.

### Mutation Ranking

Using RFECV, three antifungal drugs, including FCZ, MCF, and AmB, were ranked and predicted, respectively. The mutation ranking results are shown in **Tables 3** **–** **5**. Previously reported mutations (bolded in the table), such as Y132F, K143R, and F126L on the *ERG11*, mutations on the *TAC1B* (Rybak et al., 2020), and S639Y/S639F and S639P on the *FKS1* gene, were detected and listed as important mutations. In addition, several novel mutations were detected (marked by an asterisk). Particularly, mutations in the “hot spot” regions of the ergosterol pathway, such as I466M, G459S, and Y501H in *ERG11*, and R278H in *ERG10*, were detected. These mutations were frequently and highly ranked mutations. *FKBP12* has been reported to be associated with multiple resistance in *Candida* spp., and the S4N mutation was detected in this gene. Two frequently occurring mutations, H771R and G995S, were identified in *CDR1*, the gene encoding the ATP-Binding Cassette efflux pump transporter. Two high-frequency mutations, E49D and A18P, were also found in a specific gene (*PGA7*, *C. albicans* homolog) of *C. Auris*. These mutations should be paid special attention to in the following research.

**Table 3:** Top 20 mutations ranked by RFECV for FCZ on F1 set.

NO.	AdaBoost	GradientBoosting	DecisionTree	SVC_linear
1	**FKS1_S639P**	B9J08_003739_G672S	B9J08_003735_K325N	**ERG11_K143R**
2	FKBP12_S4N*	B9J08_003902_G126R	B9J08_003902_G126R	**ERG11_Y132F**
3	B9J08_000267_Y114D	B9J08_003902_S24P	CDR1_G995S*	ERG11_V125A
4	ERG11_Y501H*	CDR1_H771R*	ERG11_Y501H*	B9J08_004578_A202T
5	ERG11_G459S*	ERG11_Y501H*	**ERG11_K143R**	**TAC1B_K247E**
6	**ERG11_K143R**	B9J08_004467_ G22E	**TAC1B_A651T**	B9J08_004467_G22E
7	**ERG11_Y132F**	CDR1_G995S*	B9J08_004468_F82V	**ERG11_F126L**
8	B9J08_004578_M245V	**TAC1B_A651T**	**ERG11_Y132F**	B9J08_001033_L136F
9	**TAC1B_E200K**	**ERG11_K143R**	**TAC1B_F214S**	ERG11_Y501H*
10	**TAC1B_F214S**	B9J08_004468_F82V	B9J08_004467_G22E	MRR1_N647T
11	**TAC1B_K247E**	**TAC1B_F214S**	PGA7_E49D*	B9J08_004468_K506R
12	**TAC1B_A583S**	B9J08_003735_K325N	B9J08_000267_Y114D	B9J08_004818_D671N
13	**TAC1B_A651T**	**ERG11_Y132F**	UPC2_E229K	B9J08_004818_E749K
14	**TAC1B_M653V**	UPC2_E229K	**TAC1B_K247E**	B9J08_004578_E289G
15	B9J08_004468_F82V	B9J08_001033_L136F	B9J08_001033_L136F	B9J08_001030_E534K
16	PGA7_A18P*	B9J08_000267_Y114D	ERG11_I466M*	B9J08_000962_H59L
17	PGA7_E49D*	**TAC1B_K247E**	ERG10_R278H*	CDR1_E709G
18	CDR1_G995S*	B9J08_001030_E2Q	**TAC1B_M653V**	B9J08_004468_F82V
19	**TAC1B_S195C**	B9J08_003902_T917I	**FKS1_S639P**	B9J08_000961_K330N
20	**TAC1B_S192N**	PGA7_E49D*	ERG3_Y279H	FKS1_M1267I

Bolded means previously reported mutations.

*Represents drug resistance mutation should be paid special attention to.

**Table 4:** Top 20 mutations ranked by RFECV for AmB on F1 set.

NO.	AdaBoost	DecisionTree	GradientBoosting	SVC_Linear	RandomForest
1	ERG10_R278H*	ERG10_R278H*	ERG10_R278H*	ERG10_R278H*	ERG10_R278H*
2	ERG3_D283N	B9J08_003902_G126R	B9J08_003902_G126R	B9J08_003902_G126R	B9J08_003736_N36H
3	B9J08_003902_G126R	MRR1_H417L	MRR1_H417L	B9J08_005341_K374R	B9J08_003902_G126R
4	**TAC1B_S195C**	B9J08_000267_Y114D	B9J08_000267_Y114D	B9J08_000267_Y114D	B9J08_005338_V621F
5	B9J08_005341_A592D	ERG11_I466M*	ERG11_I466M*	ERG11_I466M*	B9J08_005341_N279H
6	B9J08_000962_G41E	ERG11_G459S*	ERG11_G459S*	ERG11_G459S*	CDR1_H771R*
7	**FKS1_S639Y/S639F**	**ERG11_K143R**	**ERG11_K143R**	**ERG11_K143R**	B9J08_000267_Y114D
8	FKS1_F219V	**ERG11_Y132F**	B9J08_004576_L747F	**ERG11_Y132F**	B9J08_001445_S430N
9	FKBP12_S4N*	B9J08_004818_S745P	B9J08_004818_S745P	**TAC1B_A651T**	ERG11_I466M*
10	B9J08_001033_L136F	**TAC1B_A651T**	B9J08_004818_P67H	PGA7_A18P	ERG11_G459S*
11	CDR1_G995S*	PGA7_A18P*	**TAC1B_S192N**	PGA7_E49D	**ERG11_K143R**
12	CDR1_E709G	**TAC1B_F214S**	**TAC1B_A640V**	CDR1_G995S*	**TAC1B_A651T**
13	B9J08_000267_Y114D	**TAC1B_S192N**	**TAC1B_A651T**	B9J08_005341_K918R	**TAC1B_A657V**
14	UPC2_E229K	B9J08_001445_L368F	**TAC1B_A657V**	**TAC1B_K247E**	PGA7_A18P*
15	B9J08_001445_L368F	**TAC1B_A15T**	PGA7_A18P*	**TAC1B_F214S**	PGA7_E49D*
16	B9J08_001445_V317L	**TAC1B_A583S**	B9J08_001445_L368F	**TAC1B_A657V**	B9J08_000166_V641D
17	ERG11_I466M*	B9J08_005341_A592D	**TAC1B_S195C**	B9J08_001033_L136F	**ERG11_Y132F**
18	ERG11_G459S*	PGA7_E49D*	B9J08_000268_I6F	B9J08_003735_E275G	UPC2_E229K
19	**ERG11_K143R**	ERG3_D283N	FKS1_K848R	B9J08_001030_E2Q	**TAC1B_S192N**
20	**ERG11_Y132F**	UPC2_E229K	**ERG11_Y132F**	CDR1_H771R*	B9J08_004818_S745P

Bolded means previously reported mutations.

*Represents drug resistance mutation should be paid special attention to.

**Table 5:** Top 20 mutations ranked by RFECV for MCF on F1 set.

NO.	AdaBoost	DecisionTree	GradientBoosting	SVC_Linear	RandomForest
1	B9J08_003489_D695V	**FKS1_S639Y/S639F**	**FKS1_S639Y/S639F**	B9J08_003902_G126R	**FKS1_S639Y/S639F**
2	B9J08_003726_T631S	**FKS1_S639P**	**FKS1_S639P**	**FKS1_S639Y/S639F**	**FKS1_S639P**
3	ABC_T37A	B9J08_000274_V248F	B9J08_001033_L136F	**FKS1_S639P**	B9J08_000274_F180V
4	**FKS1_S639Y/S639F**	B9J08_000274_F180V	ERG11_I466M*	CDR1_H771R*	FKBP12_S4N*
5	**FKS1_S639P**	ERG11_I466M*	B9J08_000274_V248F	ERG11_I466M*	**ERG11_Y132F**
6	FKS1_F219V	B9J08_001033_L136F	FKS1_D979N	B9J08_000274_V248G	B9J08_001033_L136F
7	CDR1_E709D	PGA7_E49D*	FKS1_K848R	FKS1_L972M	**TAC1B_A657V**
8	CDR1_V704L	**ERG11_F126L**	B9J08_000274_F180V	MRR1_R249K	ERG11_I466M*
9	UPC2_E229K	CDR1_V704L	B9J08_000274_V248G	FKBP12_S4N*	B9J08_000161_N244K
10	B9J08_000274_F180V	**TAC1B_A640V**	B9J08_003735_H8Q	B9J08_000162_D109G	ERG3_L262I
11	ERG11_I466M*	B9J08_003726_G756V	PGA7_E49D*	B9J08_000162_P110S	CDR1_V704L
12	**TAC1B_A640V**	FKS1_F219V	FKS1_S846A	MRR1_I211V	PGA7_E49D*
13	PGA7_E49D*	PGA7_A18P*	ABC_Y504H	B9J08_000274_F180V	B9J08_004009_E641K
14	B9J08_003726_G756V	B9J08_001445_L368F	B9J08_000274_N243K	B9J08_003489_D695V	FKS1_L972M
15	CDR1_E709G	ABC_V3I	MRR1_N647T	B9J08_000162_E475K	PGA7_A131T
16	B9J08_001033_L136F	B9J08_003726_N248H	B9J08_003726_K299R	**ERG11_Y132F**	**ERG11_K143R**
17	**ERG11_Y132F**	B9J08_000166_V641D	B9J08_000274_A245G	CDR1_E709D	PGA7_A18P*
18	CDR1_H771R*	B9J08_003726_S279N	**TAC1B_A640V**	B9J08_003726_P586S	FKS1_K848R
19	ABC_V3I	B9J08_000166_S685N	**ERG11_F126L**	B9J08_003622_V132L	CDR1_H771R*
20	PGA7_A18P*	B9J08_003726_K299R	B9J08_000274_A231D	B9J08_003622_S249T	ERG3_V258I

Bolded means previously reported mutations.

*Represents drug resistance mutation should be paid special attention to.

## Discussion

*C. auris* strains C1921 and C1922 sequenced in our laboratory were classified into Clade III from the phylogenetic tree, which was consistent with the tree constructed using Internal Transcribed Spacer and D1/D2 Large Ribosomal Subunit Region in the previous study (Chen et al., 2018). Previous studies classified *C. auris* into four clades: South Asia (Clade I), East Asia (Clade II), South Africa (Clade III), and South America (Clade IV) (potential fifth clade of Iranian origin), and it was emphasized that each clade has a great relationship with geographical location. The clustering results from the phylogenetic tree in this study illustrated that these strains could be divided into four clades, but the conclusion of clustering according to geographical location was not very prominent.

Machine learning technology has great potential in classifying drug resistance of strains with WGS data and analyzing high-dimensional data sets, which is very important for predicting mutations associated with drug resistance. Our model evaluation results illustrated that the machine learning classifiers performed quite different when testing different drugs. The classifier model showed excellent performance for azoles and echinocandins such as FCZ, ICZ, VCZ, and MCF, but not for others like AmB and PZ. It was speculated that there might be more information about determinants associated with azoles and echinocandins resistance but less for AmB and PZ in the three sets. This was directly indicative of the fact that the correlation between feature sets and classification targets was stronger for azoles and echinocandins, but was weaker for the two drugs. In addition, there were some deficiencies in model optimization so that only several models were optimized in the process of constructing classifier models and adjusting parameters. Therefore, optimizing models through a large number of experiments and tests should be performed in future work in order to achieve better performance.

In this study, RFECV combined with a machine learning classifier was used to predict and rank the mutations of *C. auris* related to antifungal drug resistance. In the RFECV process, different ranked mutation results were obtained by combining different classifiers. Overall, the results indicated that the RFECV method could not only rank several known mutations as important, especially for well-studied drugs but also predict some new important mutations on the genes closely related to drug resistance. Some of the predicted mutations were known to be important resistance mutations, which to some extent demonstrated the validity of our classification model. The model could obtain more reliable conclusions for well-studied drugs, such as azoles and echinocandins, while for amphotericin B, the model also predicted some resistance-related mutations. Based on these results, further research and verification are needed on the specific mutations and drug resistance mechanisms of *C. auris*.

Machine learning models can improve the prediction of important genetic mutation sites related to drug resistance in fungi, particularly beneficially for less-studied drugs. The amount of test data, or sample size, is one of the keys to the performance of machine learning methods. We speculate that 500 to 1,000 fungal samples may get satisfactory results according to previous studies. Random forest, logistic regression, and K-nearest neighbors classifier performed relatively better in this study. While in another study, PM (product-of-marginal model) and SVC-RBF ranked as the top two best-performing classifiers on MTB (Yang et al., 2018). The most common issues in machine learning lie around overfitting, underfitting, noisy data and inappropriate validation. Hence, considering all available variants and allowing machine learning methods to reduce the dimension can improve the performance. In the future, it is necessary to conduct systematic verification and related functional studies on these mutations.

This study may help to analyze the drug resistance mechanism of *C. auris*, and provide a scientific basis for developing prevention and control strategies against drug resistance and the search for possible new drug targets.

## Data Availability Statement

The datasets presented in this study can be found in online repositories. The names of the repository/repositories and accession number(s) can be found in the article/**Supplementary Material**.

## Author Contributions

LH and XC conceived the project. JZ and FC collected the samples. DL, YW, and WH conducted the NGS. YW and DL conducted the RNA analysis, analyzed data and wrote manuscript. LH evaluated all results. All authors contributed to the article and approved the submitted version.

## Funding

This study was supported by Scientific Research Project of National Natural Science Foundation of China (81971914, 81772163, 82172293), the State Key Program of National Natural Science Foundation of China (12031016), Project of Natural Science Foundation of Liaoning Province (20180550255) and Fundamental Research Funds for the Central Universities (GK201901008).

## Conflict of Interest

The authors declare that the research was conducted in the absence of any commercial or financial relationships that could be construed as a potential conflict of interest.

## Publisher’s Note

All claims expressed in this article are solely those of the authors and do not necessarily represent those of their affiliated organizations, or those of the publisher, the editors and the reviewers. Any product that may be evaluated in this article, or claim that may be made by its manufacturer, is not guaranteed or endorsed by the publisher.
