# Supplementary material for: Application of Machine Learning Classifier to Candida auris Drug Resistance Analysis
Source: Front Cell Infect Microbiol. 2021 Oct 15;11:742062. doi: 10.3389/fcimb.2021.742062 (PMC8554202; doi:10.3389/fcimb.2021.742062)
Supplement: Supplementary file 1 [file DataSheet_1.docx]

# Supplementary Material

## 1 Supplementary Data

Please see the file “resistance information.xls” for supplementary data.

## 2 Supplementary Figures and Tables


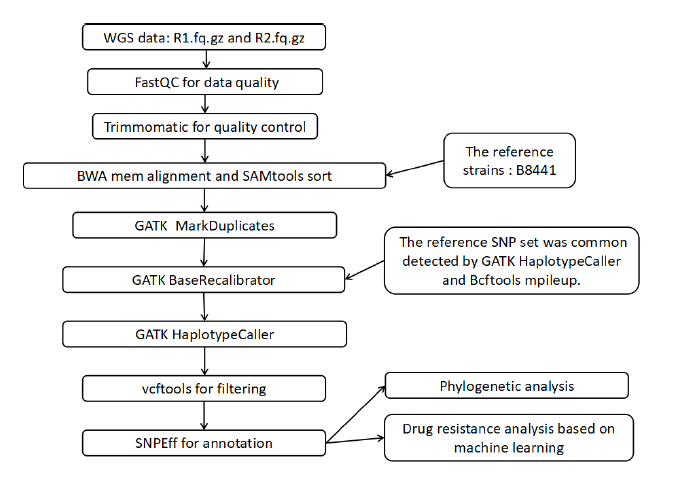


**Figure S1.** **Whole-genome sequencing data processing process.** The quality control parameter set as NexteraPE-PE.fa: 2: 30: 10 LEADING: 15 TRAILING: 15 MINLEN: 75 AVGQUAL: 20 by Trimmomatic-0.36 software. The reference strain for reads alignment was B8441. The common SNP set detected byGATK HaplotypeCaller, and Bcftools mpileup was used as the SNP reference set for BaseRecalibrator.


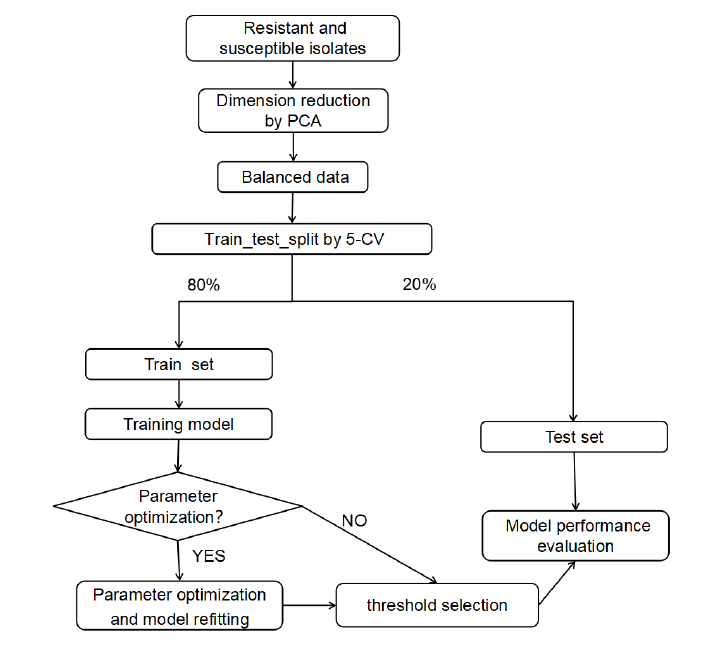


**Figure S2. Algorithmic flow based on the balanced test set**. After dimensionality reduction of PCA, the data set is directly balanced by downsampling or upsampling, and then the training set and test set are divided by the five-fold cross-validation method. The final test set obtained by the process is the test set after the balanced data set.


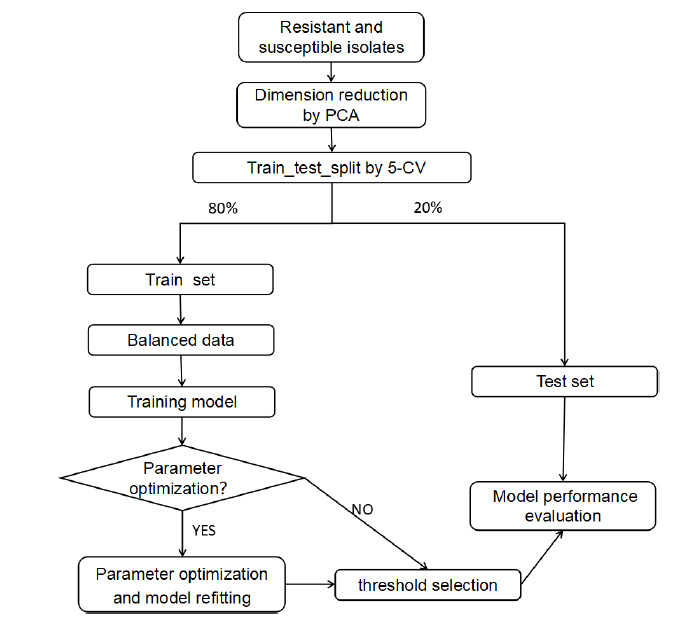


**Figure S3.** **Algorithmic flow based on the imbalanced test set.** After dimensivity reduction of PCA, the data is directly divided into a training set and test set using 5 fold cross-validation method, and then the data set is balanced on the training set by downsampling or upsampling method. The final test set obtained by this process does not go through the link of balancing the data set.


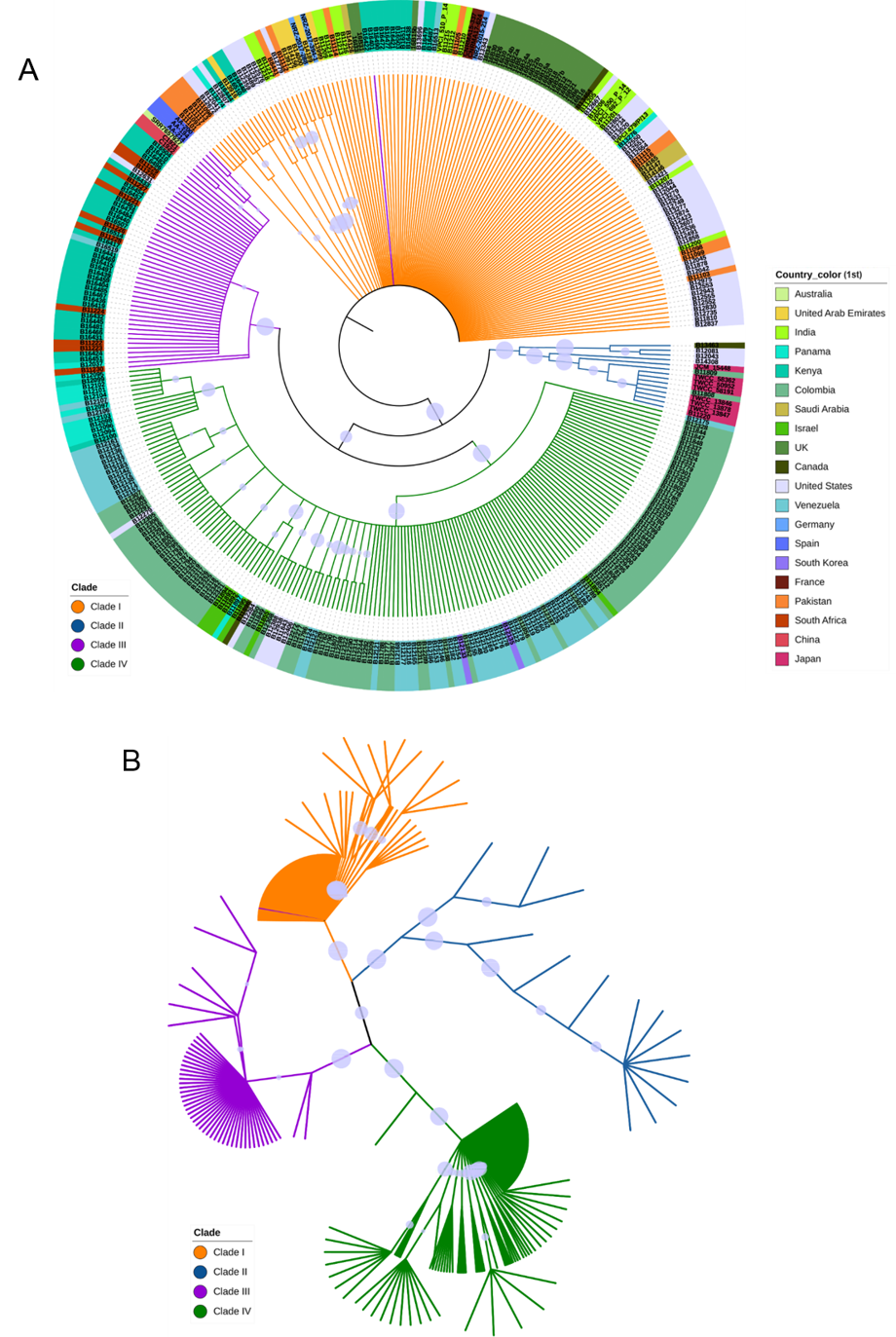


**Figure S4. Phylogenetic NJ tree of *C. auris* based on F3 set.** (A) Rooted tree. (B) Unrooted tree.

**Table S1.** SNP detection of GATK and Bcftools. The BaseRecalibrator procedure requires a known SNP set of *C. auris* as the reference; yet, no relevant published set has been available. Therefore, two software (GATK HaplotypeCaller and Bcftools mpileup) were considered for SNP detection. In this study, 246258 reference sites jointly detected by the two software were taken as the set of known SNP reference sites.

| Software | Total number | Common to both | Single sites |
| --- | --- | --- | --- |
| GATK HaplotypeCaller | 296,024 | 246,258 | 49,766 |
| Bcftools mpileup | 308,645 | 246,258 | 62,360 |

**Table S2**. A list of gene sets. The gene name and gene ID corresponding to the candidate gene set. F2 is the seven unique genes found in *C. auris*; F3 may contain the genes that determine the drug resistance, and F1 is a total gene set combining F2 and F3 genes.

| Selected gene set | Gene name | Reference B8441 geneID |
| --- | --- | --- |
| **F1** | PLB3(Candida albicans homolog) | B9J08_003486, B9J08_003621, B9J08_004010 |
|  | IFF4(Candida albicans homolog) | B9J08_000675, B9J08_001531, B9J08_004098, B9J08_004109, B9J08_004892 |
|  | PGA52(Candida albicans homolog) | B9J08_003903 |
|  | PGA26(Candida albicans homolog) | B9J08_004577 |
|  | CSA1(Candida albicans homolog) | B9J08_00195 |
|  | HYR3(Candida albicans homolog) | B9J08_004100 |
|  | PGA7(Candida albicans homolog) | B9J08_004469 |
| **F2** | FKBP12(multiple drug resistance) | B9J08_001031 |
|  | CDR1(Azoles resistance) | B9J08_000164 |
|  | ABC (Azoles resistance) | B9J08_003727,B9J08_004108 |
|  | ERG11(Azoles resistance) | B9J08_001448 |
|  | TAC1B(Azoles resistance) | B9J08_004820 |
|  | TAC1A(Azoles resistance) | B9J08_004819 |
|  | UPC2(Azoles resistance) | B9J08_000270 |
|  | MRR1(Azoles resistance) | B9J08_004061 |
|  | FKS1(Echinocandins resistance) | B9J08_000964 |
|  | FKS2(Echinocandins resistance) | B9J08_000964 |
|  | ERG2, ERG3 | B9J08_004943,B9J08_003737 |
|  | ERG5, ERG6 | B9J08_002349,B9J08_005340 |
| **F3** | F2+F3 | |

**Table S3.** Machine learning classifiers. In this paper, the machine learning model is used to classify whether *C. auris* is resistant or not. There are 10 kinds of classifiers involved, and their methods and abbreviations are shown in the following table.

| Classifiers | Summary | Abbreviations |
| --- | --- | --- |
| Logistic Regression | Logistic Regression is a widely used binary classifier, combined with a linear model and logical function, transforming the continuous values of predicted values into class 0 or class 1 in the binary classification problem. Regularization is a method to reduce the variance by punishing the complex model. In this paper, L 2 penalty term is selected to reduce the variance in the logistic regression model. | LRL2 |
| Support Vector Machine | The goal of the Support Vector Machine classifier is to find the maximum interval of dividing hyperplanes in the original sample space. If such an interval cannot be found in the original space, it is usually possible to map the original sample space to a higher dimensional space using a kernel function to find such an interval in the higher dimensional space. This paper uses the Gaussian kernel (RBF) SVC classifier and linear kernel (Linear) SVC classifier. | SVC RBF  SVC linear |
| K-Nearest Neighbors | K-nearest Neighbors classifier is a simple and widely used classifier in the supervised learning field. Given a test set, the information of K samples closest to the training set is found based on some distance measure to make a prediction. In this classification model, the voting method is often used to select the classes that appear most in the samples as the prediction results. | KNN |
| Decision Tree | The Decision Tree classifier is one of the commonly used machine learning taxonomies, usually containing a root node, several leaf nodes, and internal nodes. The root node contains the complete set of samples, each internal node corresponds to an attribute, and the leaf node corresponds to the decision result. | DT |
| Naive Bayes | Naive Bayes classifier is a specific application of Bayes’ theorem in classification. Among them, for each feature of the data, the statistical distribution of its likelihood probability must be assumed, and each feature and its likelihood probability in naive Bayes are independent of each other. This paper mainly uses Bernoulli Naive Bayes classifier and Gauss Naive Bayes classifier (GaussianNB) to carry out experiments. | BernoulliNB  GaussianNB |
| Ensemble Learning | Ensemble Learning generates several “individual learners” through a training set and combines them with a strategy that results in a model with better generalization performance than a single learner. There are three specific methods: parallelization (represented by the Random Forest), serialization (represented by AdaBoost and GradientBoosting), and Stacking. | RandomForest(RF)  AdaBoost  GradientBoosting(GB) |

**Table S4.** The number of principal components after dimension reduction with PCA method when 99% of the variance is retained.

| Data set | FCZ | AmB | MCF | VCZ | ICZ | PZ |
| --- | --- | --- | --- | --- | --- | --- |
| F1 | 30 | 30 | 30 | 13 | 11 | 10 |
| F2 | 8 | 8 | 8 | 3 | 3 | 1 |
| F3 | 29 | 29 | 28 | 15 | 13 | 12 |

**Table S5.** Clustering of *C. auris* from different countries or regions in the phylogenetic tree.

| **Clades** | **Sampling Area** |
| --- | --- |
| Clade I | India, UK, Germany, Kenya, United Arab Emirates, Saudi Arabia, Canada, United States, Pakistan |
| Clade II | Japan, United States, Canada, South Korea |
| Clade III | Kenya, Canada, Spain, South Africa, Australia, United States, China |
| Clade IV | Venezuela, Panama, Colombia, Israel, United States |

**Table S6.** Comparison of model performance under the two algorithms: AUC values on the balanced test set (table above) and AUC values on the unbalanced test set (table below). The number after ± represents the standard deviation generated after 100 repeat samplings.

| Set_Drugs | AdaBoost | BernoulliNB | DecisionTree | GaussianNB | GradientBoosting | K-NearestNeighbors | LogisticRression-L2 | RandomForest | SVC_linear | SVC_rbf |
| --- | --- | --- | --- | --- | --- | --- | --- | --- | --- | --- |
| F1_AmB | 0.9507 ± 0.0082 | 0.8606 ± 0.0261 | 0.9369 ± 0.0120 | 0.8595 ± 0.0287 | 0.9471 ± 0.0072 | 0.8998 ± 0.0110 | 0.8973 ± 0.0074 | 0.9366 ± 0.0097 | 0.8880 ± 0.0120 | 0.8776 ± 0.0162 |
| F1_MCF | 0.9964 ± 0.0004 | 0.9407 ± 0.0185 | 0.9954 ± 0.0004 | 0.9511 ± 0.0178 | 0.9946 ± 0.0004 | 0.9971 ± 0.0007 | 0.9768 ± 0.0015 | 0.9948 ± 0.0022 | 0.9878 ± 0.0027 | 0.9745 ± 0.0039 |
| F1_FCZ | 0.9871 ± 0.0072 | 0.9410 ± 0.0145 | 0.9777 ± 0.0043 | 0.9571 ± 0.0174 | 0.9903 ± 0.0053 | 0.9749 ± 0.0049 | 0.9591 ± 0.0065 | 0.9908 ± 0.0043 | 0.9673 ± 0.0071 | 0.9809 ± 0.0091 |
| F1_VCZ | 0.9566 ± 0.0041 | 0.9177 ± 0.0205 | 0.9579 ± 0.0038 | 0.9466 ± 0.0192 | 0.9550 ± 0.0071 | 0.9690 ± 0.0094 | 0.9389 ± 0.0042 | 0.9614 ± 0.0024 | 0.9073 ± 0.0027 | 0.9297 ± 0.0056 |
| F1_PZ | 0.8990 ± 0.0379 | 0.7972 ± 0.0432 | 0.9251 ± 0.0429 | 0.8023 ± 0.0492 | 0.8984 ± 0.0309 | 0.8793 ± 0.0814 | 0.8065 ± 0.0278 | 0.8700 ± 0.0349 | 0.8778 ± 0.0396 | 0.8649 ± 0.0525 |
| F1_ICZ | 0.9431 ± 0.0000 | 0.9431 ± 0.0000 | 0.9500 ± 0.0000 | 0.9425 ± 0.0030 | 0.9500 ± 0.0000 | 0.9651 ± 0.0099 | 0.9385 ± 0.0048 | 0.9500 ± 0.0000 | 0.9541 ± 0.0021 | 0.9423 ± 0.0002 |
| F2_AmB | 0.8373 ± 0.0203 | 0.7465 ± 0.0012 | 0.8229 ± 0.0165 | 0.7913 ± 0.0057 | 0.8185 ± 0.0255 | 0.8719 ± 0.0249 | 0.7843 ± 0.0104 | 0.8379 ± 0.0106 | 0.7749 ± 0.0178 | 0.7818 ± 0.0023 |
| F2_MCF | 0.8807 ± 0.0201 | 0.8138 ± 0.0349 | 0.8216 ± 0.0079 | 0.7314 ± 0.0188 | 0.8546 ± 0.0190 | 0.9648 ± 0.0127 | 0.7566 ± 0.0094 | 0.7927 ± 0.0170 | 0.7666 ± 0.0208 | 0.7994 ± 0.0297 |
| F2_FCZ | 0.9621 ± 0.0048 | 0.8775 ± 0.0087 | 0.9409 ± 0.0040 | 0.9327 ± 0.0178 | 0.9579 ± 0.0083 | 0.9429 ± 0.0051 | 0.9512 ± 0.0091 | 0.9555 ± 0.0077 | 0.9204 ± 0.0068 | 0.9364 ± 0.0285 |
| F2_VCZ | 0.9024 ± 0.0074 | 0.9152 ± 0.0136 | 0.9250 ± 0.0107 | 0.8829 ± 0.0145 | 0.9136 ± 0.0086 | 0.9017 ± 0.0394 | 0.9381 ± 0.0025 | 0.8735 ± 0.0033 | 0.8781 ± 0.0022 | 0.9111 ± 0.0050 |
| F2_PZ | 0.7081 ± 0.0479 | 0.7338 ± 0.0627 | 0.6191 ± 0.0156 | 0.6953 ± 0.0448 | 0.6735 ± 0.0591 | 0.6199 ± 0.0658 | 0.6394 ± 0.0005 | 0.7872 ± 0.0605 | 0.7051 ± 0.0278 | 0.7041 ± 0.0208 |
| F2_ICZ | 0.9874 ± 0.0032 | 0.9210 ± 0.0000 | 0.9210 ± 0.0000 | 0.9500 ± 0.0000 | 0.9500 ± 0.0000 | 0.9500 ± 0.0000 | 0.9500 ± 0.0000 | 0.9500 ± 0.0000 | 0.9500 ± 0.0000 | 0.9210 ± 0.0000 |
| F3_AmB | 0.8647 ± 0.0159 | 0.7982 ± 0.0154 | 0.8828 ± 0.0241 | 0.8262 ± 0.0281 | 0.8784 ± 0.0193 | 0.8773 ± 0.0286 | 0.8807 ± 0.0284 | 0.9026 ± 0.0285 | 0.8374 ± 0.0262 | 0.8306 ± 0.0342 |
| F3_MCF | 0.9870 ± 0.0054 | 0.9090 ± 0.0196 | 0.9678 ± 0.0048 | 0.9377 ± 0.0099 | 0.9883 ± 0.0040 | 0.9818 ± 0.0073 | 0.9735 ± 0.0028 | 0.9914 ± 0.0044 | 0.9781 ± 0.0113 | 0.9424 ± 0.0057 |
| F3_FCZ | 0.9740 ± 0.0115 | 0.9347 ± 0.0258 | 0.9565 ± 0.0098 | 0.8948 ± 0.0186 | 0.9698 ± 0.0116 | 0.9227 ± 0.0068 | 0.9556 ± 0.0139 | 0.9787 ± 0.0076 | 0.9609 ± 0.0181 | 0.9689 ± 0.0126 |
| F3_VCZ | 0.9485 ± 0.0056 | 0.9153 ± 0.0190 | 0.9196 ± 0.0138 | 0.9175 ± 0.0100 | 0.9395 ± 0.0035 | 0.9370 ± 0.0173 | 0.9083 ± 0.0028 | 0.9046 ± 0.0100 | 0.9424 ± 0.0055 | 0.9321 ± 0.0129 |
| F3_PZ | 0.7562 ± 0.0397 | 0.7978 ± 0.0269 | 0.7409 ± 0.0262 | 0.6182 ± 0.0918 | 0.8677 ± 0.0332 | 0.8424 ± 0.0506 | 0.7926 ± 0.0230 | 0.8919 ± 0.0472 | 0.7083 ± 0.0161 | 0.8079 ± 0.0415 |
| F3_ICZ | 0.9431 ± 0.0000 | 0.9701 ± 0.0014 | 0.9431 ± 0.0000 | 0.9425 ± 0.0030 | 0.9431 ± 0.0000 | 0.9498 ± 0.0009 | 0.9439 ± 0.0017 | 0.9500 ± 0.0000 | 0.9459 ± 0.0004 | 0.9388 ± 0.0002 |

| Set_Drugs | AdaBoost | BernoulliNB | DecisionTree | GaussianNB | GradientBoosting | K-NearestNeighbors | LogisticRression-L2 | RandomForest | SVC_linear | SVC_rbf |
| --- | --- | --- | --- | --- | --- | --- | --- | --- | --- | --- |
| F1_AmB | 0.8924 ± 0.0202 | 0.8090 ± 0.0893 | 0.8692 ± 0.0193 | 0.8139 ± 0.0214 | 0.8992 ± 0.0128 | 0.8556 ± 0.0053 | 0.8800 ± 0.0069 | 0.9136 ± 0.0144 | 0.8419 ± 0.0102 | 0.8429 ± 0.0322 |
| F1_MCF | 0.9626 ± 0.0457 | 0.9568 ± 0.0370 | 0.9680 ± 0.0398 | 0.9187 ± 0.0499 | 0.9664 ± 0.0404 | 0.9345 ± 0.0399 | 0.9669 ± 0.0351 | 0.9733 ± 0.0260 | 0.9739 ± 0.0237 | 0.9807 ± 0.0162 |
| F1_FCZ | 0.9404 ± 0.0082 | 0.9415 ± 0.0100 | 0.9331 ± 0.0130 | 0.9126 ± 0.0182 | 0.9572 ± 0.0034 | 0.9423 ± 0.0069 | 0.9413 ± 0.0038 | 0.9593 ± 0.0043 | 0.9397 ± 0.0037 | 0.9475 ± 0.0047 |
| F1_VCZ | 0.9098 ± 0.0580 | 0.9223 ± 0.0620 | 0.8478 ± 0.0378 | 0.8681 ± 0.0633 | 0.9112 ± 0.0518 | 0.8894 ± 0.0595 | 0.8641 ± 0.0478 | 0.9441 ± 0.0351 | 0.8721 ± 0.0777 | 0.8790 ± 0.0195 |
| F1_PZ | 0.7159 ± 0.0130 | 0.7523 ± 0.0252 | 0.7663 ± 0.0814 | 0.6683 ± 0.0496 | 0.7403 ± 0.0244 | 0.7135 ± 0.0152 | 0.7548 ± 0.0464 | 0.7846 ± 0.0270 | 0.6854 ± 0.0474 | 0.6665 ± 0.0407 |
| F1_ICZ | 0.9630 ± 0.0114 | 0.9630 ± 0.0114 | 0.9630 ± 0.0114 | 0.9630 ± 0.0114 | 0.9630 ± 0.0114 | 0.9696 ± 0.0168 | 0.9612 ± 0.0122 | 0.9630 ± 0.0114 | 0.9695 ± 0.0134 | 0.9631 ± 0.0116 |
| F2_AMB | 0.8007 ± 0.0031 | 0.7357 ± 0.0124 | 0.7970 ± 0.0032 | 0.7759 ± 0.0106 | 0.8008 ± 0.0033 | 0.7973 ± 0.0103 | 0.7854 ± 0.0105 | 0.8008 ± 0.0033 | 0.7686 ± 0.0189 | 0.7815 ± 0.0120 |
| F2_MCF | 0.7479 ± 0.0192 | 0.6266 ± 0.0538 | 0.7486 ± 0.0184 | 0.7085 ± 0.0270 | 0.7565 ± 0.0179 | 0.6017 ± 0.0533 | 0.5697 ± 0.0316 | 0.7538 ± 0.0185 | 0.5182 ± 0.0433 | 0.7089 ± 0.0211 |
| F2_FCZ | 0.9233 ± 0.0054 | 0.8908 ± 0.0087 | 0.9302 ± 0.0092 | 0.9033 ± 0.0084 | 0.9279 ± 0.0102 | 0.9205 ± 0.0105 | 0.9153 ± 0.0100 | 0.9314 ± 0.0095 | 0.8990 ± 0.0224 | 0.9117 ± 0.0081 |
| F2_VCZ | 0.9075 ± 0.0482 | 0.9070 ± 0.0525 | 0.9066 ± 0.0563 | 0.9107 ± 0.0451 | 0.8980 ± 0.0677 | 0.8842 ± 0.0738 | 0.9136 ± 0.0498 | 0.9077 ± 0.0524 | 0.9049 ± 0.0487 | 0.8945 ± 0.0420 |
| F2_PZ | 0.6322 ± 0.0176 | 0.6301 ± 0.0155 | 0.6330 ± 0.0180 | 0.6364 ± 0.0182 | 0.6309 ± 0.0176 | 0.6199 ± 0.0658 | 0.6595 ± 0.0296 | 0.6300 ± 0.0164 | 0.6425 ± 0.0240 | 0.6226 ± 0.0674 |
| F2_ICZ | 0.9369 ± 0.0121 | 0.9375 ± 0.0112 | 0.9356 ± 0.0142 | 0.9375 ± 0.0112 | 0.9375 ± 0.0112 | 0.9375 ± 0.0112 | 0.9375 ± 0.0112 | 0.9375 ± 0.0112 | 0.9375 ± 0.0112 | 0.9375 ± 0.0112 |
| F3_AmB | 0.7524 ± 0.0307 | 0.7824 ± 0.0471 | 0.7556 ± 0.0181 | 0.7747 ± 0.0248 | 0.7656 ± 0.0164 | 0.7536 ± 0.0176 | 0.7700 ± 0.0173 | 0.8116 ± 0.0244 | 0.7032 ± 0.0182 | 0.7197 ± 0.0173 |
| F3_MCF | 0.8962 ± 0.0187 | 0.8735 ± 0.0259 | 0.8746 ± 0.0201 | 0.8801 ± 0.0307 | 0.9165 ± 0.0218 | 0.8943 ± 0.0122 | 0.9510 ± 0.0089 | 0.9239 ± 0.0128 | 0.9077 ± 0.0103 | 0.9349 ± 0.0107 |
| F3_FCZ | 0.9149 ± 0.0174 | 0.9058 ± 0.0144 | 0.9066 ± 0.0164 | 0.8738 ± 0.0245 | 0.9347 ± 0.0120 | 0.9326 ± 0.0106 | 0.9280 ± 0.0108 | 0.9531 ± 0.0090 | 0.9219 ± 0.0184 | 0.9338 ± 0.0136 |
| F3_VCZ | 0.8495 ± 0.0371 | 0.9299 ± 0.0326 | 0.8026 ± 0.0184 | 0.8904 ± 0.0419 | 0.8819 ± 0.0238 | 0.8680 ± 0.0609 | 0.8726 ± 0.0363 | 0.9058 ± 0.0261 | 0.9434 ± 0.0300 | 0.8841 ± 0.0322 |
| F3_PZ | 0.6565 ± 0.0247 | 0.6574 ± 0.0657 | 0.6620 ± 0.0329 | 0.6275 ± 0.0692 | 0.6483 ± 0.0379 | 0.6313 ± 0.0200 | 0.6411 ± 0.0559 | 0.6737 ± 0.0346 | 0.6009 ± 0.0118 | 0.5967 ± 0.0117 |
| F3_ICZ | 0.9630 ± 0.0114 | 0.9657 ± 0.0144 | 0.9630 ± 0.0114 | 0.9630 ± 0.0114 | 0.9630 ± 0.0114 | 0.9599 ± 0.0080 | 0.9648 ± 0.0113 | 0.9630 ± 0.0114 | 0.9637 ± 0.0102 | 0.9630 ± 0.0114 |
